# Relationship Between Invasive Fungal Infection and Hypostatic Pneumonia: A Prospective Cohort Study

**DOI:** 10.3389/fmicb.2022.859359

**Published:** 2022-06-20

**Authors:** Lin Liu, Chang Liu, Jianrong Cai, Jiayun Chen, Jie Chen, Yuanyuan Fu, Kexin Yi, Hui Wang, Xue Li

**Affiliations:** ^1^School of Public Health, Shanghai Jiao Tong University School of Medicine, Shanghai, China; ^2^Department of Ultrasound, Chongming Branch, Xinhua Hospital Affiliated to Shanghai Jiao Tong University School of Medicine, Shanghai, China

**Keywords:** invasive fungal infection, hypostatic pneumonia, 1, 3-β-D-glucan, prospective cohort study, globulin, C-reactive protein

## Abstract

**Background:**

The short-term mortality of hypostatic pneumonia (HP) is very high, and the treatment outcome is poor. The clinical diagnosis and treatment are primarily focused on bacterial and viral infection, ignoring the role of fungal infection at present. This study aims to validate the relationship between Invasive Fungal Infections (IFI) and HP.

**Methods:**

In the cross-sectional study, a total of 11,371 participants have been enrolled. In the prospective cohort study, 4,441 individuals have been included at baseline and followed up from 2015 to 2019 with a total person years of 8,484.65. The standard procedures were used to assess questionnaire investigations, laboratory testing, and anthropometric indicators. For data analysis, logistic regression, restricted cubic spline, log-rank regression, Cox regression, and linear mixed effects model were applied to assess the relationship between IFI and HP risk longitudinally.

**Results:**

In the cross-sectional study, elevated β-D-Glucan (BDG) concentrations are associated with a higher risk of HP prevalence in the total population, men, and women (*OR*_*T*3 *vs*_._*T*1_ [95% *CI*s]: 2.12 [1.55, 2.91]; 2.01 [1.35, 2.99]; 2.34 [1.39, 3.94]), which were verified by a dose–effect relationship in the restricted cubic spline model. In the cohort study, Cox and Log-rank regression showed that the elevated BDG concentrations are associated with a significantly higher risk of HP incidence than participants with lower BDG concentrations (*HR*_*T*3 *vs*_._T1_ [95% *CIs*]: 2.72 [1.36, 5.43], *p*_*Log*–*rank*_ = 0.0086). During 5 years, the globulin (GLB) and C-reactive protein (CRP) were always on the top in the highest category of BDG concentrations. Between low and high BDG concentration, the total trend of GLB concentration was falling and the total trend of CRP concentration was rising with the increase of years (all the *p*-values < 0.0001).

**Conclusion:**

In this study, IFI is associated with a higher risk of HP, with time sequence and related mechanisms requiring further investigation in the future.

## Introduction

Hypostatic pneumonia (HP) is a slow-developing chronic pulmonary disorder. It is primarily inflamed by chronic congestion, blood stasis, and edema at the bottom of the lungs of long-term bed-ridden patients (Capel, 1976). At present, few studies have been undertaken to investigate the comprehensive classification of HP. A central HP incidence density in elderly bed-ridden patients can reach 13.9/1,000 person-days (Jiao et al., 2019). Without timely and appropriate treatment, patients with HP will die of multiple organ failure (Traver, 1974). It was reported that pneumonia causes 68.1% of deaths in bed-ridden patients within half a year, and the 3-month mortality of bed-ridden patients with merely one of the leading HP was 16.56% (Kosaka et al., 2012; Jiao et al., 2021). According to current standard practice, pathology specimens of patients with HP are assessed for the presence of bacteria or viruses, paying little attention to other pathogens (Koechlin et al., 2014; Song et al., 2021). However, the effect of prevention and treatment is poor, so whether there are other risk factors or dominant causes is unclear and needs to be studied (Shi et al., 2019; Chen et al., 2021).

Invasive fungal infection (IFI) refers to the invasion of organ tissue, blood, or other sterile body sites by fungi (Webb et al., 2018). Lung is one of the main target organs of IFIs (Luo et al., 2010), with symptoms such as cough, fever, hemoptysis, and dyspnea, similar to bacterial pneumonia (Luo et al., 2010; Dion and Ashurst, 2021; Njovu et al., 2021). Pulmonary mycosis is self-limiting generally, but several patients will evolve to develop acute or chronic infection (Di Mango et al., 2019). Until now, most clinicians have focused on bacterial and viral causes of HP. However, fungal pathogens are often ignored (Koechlin et al., 2014; Song et al., 2021). Fungi and bacteria interact with different mechanisms when linked with HP development. Recently, fungi have been reported to increase bacteria pneumonia prevalence in rats by hindering reactive oxygen species (ROS) production in alveolar macrophages (Roux et al., 2009). In contrast, the biofilm formed by fungi can increase the number of bacteria contributing to the development of pneumonia (Luo et al., 2021). In addition, the prolonged-time bed-ridden increases the probability of pulmonary fungal infection (Yuan et al., 2021). Although reports of the interaction between fungi and bacteria have been conflicted within *in vitro* and *in vivo* experiments (Dhamgaye et al., 2016), it is unknown whether fungal infection contributes to HP and the coinfection mechanism in humans. Therefore, fundamental questions need to be addressed to develop an optimized treatment scheme for HP and reduce mortality if IFI plays an important role. Whether IFI leads to an increased risk of HP incidence with time sequence-verified by epidemiological evidence, mainly previously considered a bacterial or viral infection (Koechlin et al., 2014; Song et al., 2021)?

In this study, a cross-sectional study was initially performed with a large sample size, combined with a longitudinal study to analyze the time sequence between IFI and the risk of HP incidence.

## Materials and Methods

### Cross-Sectional and Prospective Cohort Study

#### Subjects

**FIGURE 1:**
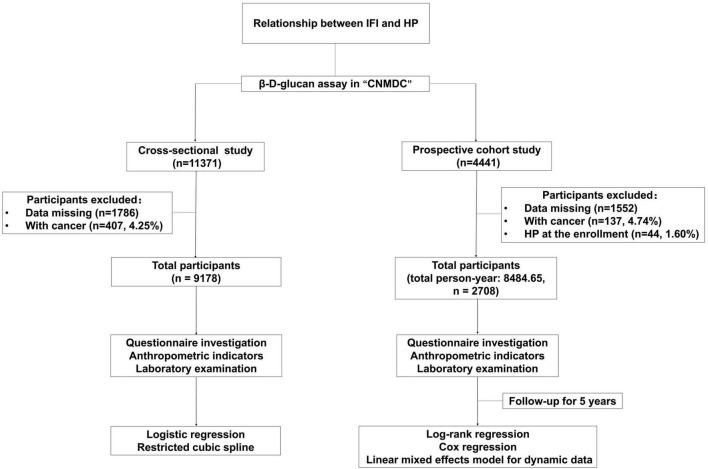
Flow chart for the cross-sectional and prospective cohort study. IFI, invasive fungal infection; HP, hypostatic pneumonia.

All the participants were recruited from “A Multi-Center Cohort Study for Nutrition, Metabolism, and Diabetic angiopathies in the Chinese population by Multi-omics method (CNMDC) ChiCTR1800018631.” The screening process is detailed in Figure 1. For the cross-sectional study, a total of 11,371 subjects have been enrolled, and all the participants included were tested by a β-D-Glucan (BDG) Assay for IFI. The exclusion criteria were as follows: Participants with cancer (407, 4.25%) and data missing. After screening, the total number of the final target population is 9,178. In the 5-years dynamic cohort study, a total of 4,441 participants with results on BDG Assay for IFI have been included from 2015. Exclusion criteria were as follows: Participants with cancer (137, 4.74%), with HP at baseline (44, 1.60%) and data missing. The total number of the final target population in the cohort study is 2,708. This study was approved by the ethics committee of Xinhua Hospital Chongming Branch, affiliated with Shanghai Jiao Tong University School of Medicine.

#### Data Collection

The demographic information was collected using questionnaire. Body mass index (BMI) was calculated by height and weight which were measured by physicians. Systolic blood pressure (SBP) and diastolic blood pressure (DBP) were measured after sitting for 10 min. Blood samples were collected after fasting for 12 h. The concentrations of BDG, serum uric acid (SUA), creatinine (Cr), total cholesterol (TC), triglyceride (TG), low-density lipoprotein cholesterol (LDL-C), high-density lipoprotein cholesterol (HDL-C), Apolipoprotein A1 (Apo A1), Apolipoprotein B (Apo B), total protein (TP), globulin (GLB), C-reactive protein (CRP), and fasting blood glucose (FBG) were determined using standard laboratory procedures. All assays were conducted using an AU5800 clinical chemistry analyzer (Corp. Beckman Coulter, United States). The diagnoses of HP were according to the criteria as follows by clinicians: a white blood cell count > 10 × 10^9^/L or < 4 × 10^9^/L; clinical symptoms and a chest X-ray examination (Song et al., 2021).

#### Statistical Analyses

In the cross-sectional and cohort studies, participants were divided into three groups according to BDG concentration tertile and presented as T1, T2, and T3. The descriptive data were expressed as mean ($\bar{\text{X}}$) and standard deviation (*SD*) or numbers and percentages (%). One-way analysis of variance was used to ascertain the significance of differences between continuous variables (Ntaios et al., 2012). The chi-square test was used to test for differences in categorical variables (Ntaios et al., 2012). A logistic regression model (Wu et al., 2022) was used to assess the odds ratio (*OR*) and 95% confidence interval (*CI*) of HP among BDG tertiles. A restricted cubic spline (Greenland, 1995) was used to estimate the possible relationship between the OR and BDG concentration with three knots located at the distributions’ 25th, 50th, and 75th percentiles. Log-rank regression and Cox regression (Schober and Vetter, 2021) were used to test the effect of IFI on HP risk. Linear mixed effects model was used to analyze the slope of variables with repeated observations (Roy, 2006). All statistical tests were performed using a two-sided test using SAS 9.4, and a *p*-value < 0.05 was considered statistically significant.

## Results

### The Cross-Sectional Study

#### The Baseline Clinical Characteristics of the Study Population With or Without Hypostatic Pneumonia in the Cross-Sectional Study

The total participant population was 11,371. After excluding those subjects with missing data (1,786, 15.71%) and then eliminating participants who had cancer (407, 4.25%), a total of 9,178 individuals were retained in the study, comprising 5,337 men and 3,841 women. The proportion of IFI in HP patients (41.21%) was much greater than that of the control population (27.96%). The BDG tertiles’ cut-off value was the same as previous research of IFI on coronary heart disease (CHD).

Table 1 shows the baseline clinical characteristics of the subjects with or without HP in the cross-sectional study. The two groups have significant differences in gender proportion, age, SBP, DBP, TC, LDL-C, HDL-C, Apo A1, Apo B, TP, GLB, and SUA. Men were more likely to be diagnosed with HP, and age and GLB were higher in HP. Meanwhile, SBP, DBP, TC, LDL-C, HDL-C, Apo A1, Apo B, TP, and SUA were lower in HP. There were no significant differences in BMI, TG, and Cr.

**TABLE 1:** Characteristics of hypostatic pneumonia (HP) and non-HP participants in the cross-sectional study.

	HP	Non-HP	*P*-value
N, %	313 (3.41)	8,865 (96.59)	
Male, %	199 (3.73)	5,138 (96.27)	0.0476
Age (years)	78.97 ± 10.55	73.71 ± 13.46	<0.0001
BMI (kg/m^2^)	24.88 ± 0.68	25.17 ± 10.86	0.6355
SBP (mmHg)	131.36 ± 17.68	134.88 ± 15.71	0.0001
DBP (mmHg)	76.23 ± 7.84	77.87 ± 8.48	0.0007
FBG (mmol/L)	6.95 ± 3.07	6.92 ± 2.86	0.8790
TC (mmol/L)	3.77 ± 1.23	4.31 ± 1.15	<0.0001
TG (mmol/L)	1.04 ± 0.68	1.00 ± 0.60	0.3206
LDL-C (mmol/L)	1.95 ± 0.88	2.28 ± 0.83	<0.0001
HDL-C (mmol/L)	1.28 ± 0.45	1.62 ± 0.55	<0.0001
Apo A1 (g/L)	0.85 ± 0.23	1.08 ± 0.30	<0.0001
Apo B (g/L)	0.71 ± 0.28	0.76 ± 0.24	0.0002
TP (g/L)	60.26 ± 7.53	64.54 ± 7.11	<0.0001
GLB (g/L)	30.67 ± 6.53	29.29 ± 5.67	<0.0001
UA (μmol/L)	260.03 ± 136.41	288.74 ± 113.51	<0.0001
Cr (μmol/L)	83.88 ± 71.29	107.83 ± 453.21	0.3500

#### The Association Between β-D-Glucan Concentration and Hypostatic Pneumonia Risk in the Cross-Sectional Study

**FIGURE 2:**
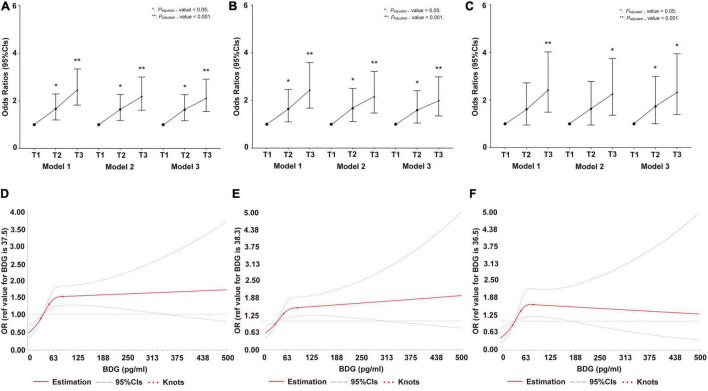
Invasive fungal infection (IFI) on hypostatic pneumonia (HP) risk in cross-sectional study. **(A)** The odds ratio (*OR*) and 95% *CIs* of IFI on HP risk in total population. **(B)** The *OR* and 95% *CIs* of IFI on HP risk in men. **(C)** The *OR* and 95% *CIs* of IFI on HP risk in women. **(D)** Restricted cubic spline of association between β-D-Glucan (BDG) concentration and *OR* (95% *CIs*) of IFI on HP risk in total population. **(E)** Restricted cubic spline of association between BDG concentration and *OR* (95% *CIs*) of IFI on HP risk in men. **(F)** Restricted cubic spline of association between BDG concentration and *OR* (95% *CIs*) of IFI on HP risk in women. In **(A–F)**, Model 1: adjusted by age and gender (only in total population); Model 2: adjusted by age, gender (only in total population), BMI, SBP, diastolic blood pressure (DBP), FBG, TC, TG, HDL-C, Apo-B, UA, and Cr; Model 3: adjusted by age (only in total population), gender, BMI, SBP, DBP, FBG, TC, TG, HDL-C, Apo-B, UA, Cr, LDL-C, Apo-A1, and TP. BMI, body mass index; SBP, systolic blood pressure; DBP, diastolic blood pressure; FBG, fasting blood glucose; TC, total cholesterol; TG, triglyceride; HDL-C, high-density lipoprotein cholesterol; Apo B, Apolipoprotein B; UA, uric acid; Cr, creatinine; LDL-C, low-density lipoprotein cholesterol; Apo A1, Apolipoprotein A1; TP, total protein; OR, odds ratio; CIs, confidence intervals, IFI, invasive fungal infection; HP, hypostatic pneumonia.

The logistic regression model revealed that high BDG concentration was independently associated with HP risk in all participants, men, and women (Figures 2A–C). In the restricted cubic spline analysis, a significant *J*-shape relationship was shown in Figures 2D–F, with a greater risk of HP at higher BDG concentrations referenced to lower ones in all participants, men, and women. The confounding factors of the above two models included age, gender (only in total population), BMI, SBP, DBP, FBG, TC, TG, LDL-C, HDL-C, Apo-A1, Apo-B, TP, UA, and Cr.

### The Prospective Cohort Study

#### The Baseline Clinical Characteristics of the Study Population Categorized According to the Serum β-D-Glucan Concentration in the Cohort Study

Four thousand four hundred forty-one individuals of the total population were followed up for 5 years. After excluding the subjects with missing data (1,552, 34.95%), then eliminating those participants also diagnosed with cancer (137, 4.74%), and screening out who had HP at the time of enrollment (44, 1.60%), a total of 2,708 individuals (total person year 8,484.65, January 2015 to December 2019) were analyzed, including 77 with newly diagn osed HP.

Table 2 shows the baseline clinical characteristics of the subjects categorized according to their BDG concentrations in the cohort study. Among the three tertiles, there are significant differences in FBG, TP, GLB, and SUA. The higher the BDG concentration, the lower the SUA concentration, with opposite trends for TP and GLB. FBG was the lowest in the moderate BDG group. There are no significant differences in gender proportion, age, BMI, SBP, DBP, TC, TG, LDL-C, HDL-C, Apo A1, Apo B, and Cr.

**TABLE 2:** Characteristics of participants according to β-D-Glucan (BDG) concentration at baseline in the prospective cohort study.

	BDG concentration (pg/mL)			
	T 1 < 38.0	T 2 38.0–72.5	T 3 ≥ 72.5	*P*-value
N, %	893 (32.98)	919 (33.94)	896 (33.09)	
Male, %	508 (32.09)	545 (34.43)	530 (33.48)	0.3315
Age (years)	74.94 ± 11.37	75.32 ± 11.19	75.22 ± 10.47	0.7435
BMI (kg/m^2^)	25.19 ± 9.58	24.87 ± 2.42	24.97 ± 7.93	0.6321
SBP (mmHg)	137.25 ± 16.98	136.85 ± 18.32	136.24 ± 17.37	0.4708
DBP (mmHg)	78.34 ± 9.16	78.10 ± 9.08	77.34 ± 9.31	0.0564
FBG (mmol/L)	7.05 ± 2.84	7.01 ± 2.80	7.33 ± 3.11	0.0416
TC (mmol/L)	4.19 ± 1.14	4.23 ± 1.14	4.27 ± 1.20	0.2847
TG (mmol/L)	1.00 ± 0.59	0.99 ± 0.64	0.94 ± 0.53	0.0782
LDL-C (mmol/L)	2.20 ± 0.83	2.23 ± 0.83	2.24 ± 0.86	0.4425
HDL-C (mmol/L)	1.66 ± 0.56	1.64 ± 0.56	1.63 ± 0.57	0.3539
Apo A1 (g/L)	1.10 ± 0.29	1.08 ± 0.31	1.08 ± 0.33	0.2376
Apo B (g/L)	0.74 ± 0.28	0.73 ± 0.22	0.75 ± 0.24	0.1108
TP (g/L)	64.01 ± 6.74	64.81 ± 7.20	65.45 ± 7.71	0.0001
GLB (g/L)	28.21 ± 5.08	29.28 ± 5.56	31.26 ± 6.15	< 0.0001
UA (μmol/L)	302.06 ± 117.29	292.54 ± 118.05	288.16 ± 118.38	0.0389
Cr (μmol/L)	100.32 ± 266.93	88.75 ± 185.69	106.76 ± 292.87	0.3041

#### The Association Between β-D-Glucan Concentration and Hypostatic Pneumonia Risk in the Cohort Study

**FIGURE 3:**
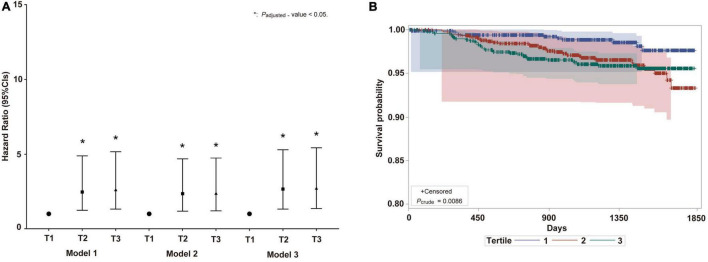
The IFI on HP risk in prospective cohort study. **(A)** Hazard ratio and 95% *CIs* of IFI on HP risk in total population. Model 1: adjusted by age and gender; Model 2: adjusted by age, gender, BMI, SBP, DBP, FBG, TC, TG, HDL-C, Apo-B, UA, and Cr; Model 3: adjusted by age, gender, BMI, SBP, DBP, FBG, TC, TG, HDL-C, Apo-B, UA, Cr, LDL-C, Apo-A1, and TP. BMI, body mass index; SBP, Systolic blood pressure; DBP, diastolic blood pressure; FBG, fasting blood glucose; TC, total cholesterol; TG, triglyceride; HDL-C, high-density lipoprotein cholesterol; Apo B, Apolipoprotein B; UA, uric acid; Cr, creatinine; LDL-C, low-density lipoprotein cholesterol; Apo A1, Apolipoprotein A1; TP, total protein; OR, odds ratio. **(B)** Survival probability curve of HP among BDG concentration analyzed by log-rank regression. CIs, confidence intervals; IFI, invasive fungal infection; HP, hypostatic pneumonia. The meaning of “*” is *P*-value < 0.05.

The overall population in the highest and moderate categories of BDG concentration had the lowest HP survival probabilities (*p* = 0.0086) (Figure 3B). The Cox regression model showed that the high BDG concentrations are independently associated with HP in all participants (Figure 3A). Subgroup analysis by gender was not performed due to the limited number of HP cases. The GLB and CRP were always on the top in the highest category of BDG concentrations during 5 years (Table 3). There were significant differences in the changing trends of GLB concentration between the low and high BDG concentration groups (Slope_*control, elevated*_: −0.67 vs. −0.685, *p* < 0.0001) and CRP concentration (Slope_*control, elevated*_: 1.771 vs. 1.835, *p* < 0.0001) with increasing years. The confounding factors of the Cox models included age, gender, BMI, SBP, DBP, FBG, TC, TG, LDL-C, HDL-C, Apo-A1, Apo-B, TP, UA, and Cr.

**TABLE 3:** The changes of globulin (GLB) and C-reactive protein (CRP) concentration between the follow-up years according to BDG concentration.

Indexes	BDG	2015	2016	2017	2018	2019	^#^*P*-value
GLB (g/L)	T1	31.68 ± 4.12	28.78 ± 5.30	26.98 ± 5.25	26.79 ± 4.95	29.48 ± 4.97	<0.0001
	T2	31.71 ± 5.23	30.46 ± 6.32	28.39 ± 5.42	28.50 ± 5.68	29.47 ± 4.79	<0.0001
	T3	33.63 ± 6.06	32.66 ± 6.89	28.39 ± 5.43	30.49 ± 6.59	31.29 ± 6.25	<0.0001
	*P -value	0.1122	<0.0001	0.0023	<0.0001	0.0002	
CRP (mg/L)	T1	14.92 ± 20.22	33.34 ± 47.12	29.80 ± 44.76	20.92 ± 30.19	27.06 ± 35.41	0.0277
	T2	18.09 ± 3.00	28.78 ± 37.76	26.63 ± 33.64	25.32 ± 31.32	32.86 ± 39.97	0.0504
	T3	30.98 ± 41.99	40.07 ± 46.38	47.24 ± 50.09	40.46 ± 45.23	39.96 ± 47.06	0.0262
	*P -value	0.1163	0.0212	< 0.0001	<0.0001	0.0013	

*Data were indicated as $\bar{\text{X}}$ ± SD.*

*P-value: the difference between BDG tertile groups in the same year by Analysis of Variance (ANOVA) tests.

*^#^P-value: the difference between years in the same BDG tertile groups by ANOVA tests.*

## Discussion

In this study, the dose–effect relationship was confirmed using restricted cubic spline analysis in the cross-sectional study. Elevated BDG concentrations increased the risk of HP incidence with the time sequence in the prospective cohort study. This study is the first and largest population study focusing on the relationship between IFI and the development of HP incidence in humans.

Previously, it was considered that HP was primarily due to bacteria and viruses. However, the current study is first analyzed the effect of IFI on the development of HP in a cross-sectional and prospective cohort study. Serum BDG concentration in humans is a biomarker for detecting IFI and is a mycological criterion in the European Organization for Research and Treatment of Cancer/Mycoses Study Group (EORTC/MSG) (De Pauw et al., 2008; Karageorgopoulos et al., 2011). Using the continuous variable of BDG concentrations is better than IFI to analyze whether IFI is associated with the elevated HP risk with time sequences. In the cross-sectional study, the odds ratios are raised with increasing BDG concentrations, indicating that IFI increased the risk of HP prevalence, consistently observed separately in men and women. Moreover, the restricted cubic spline showed that the association between BDG concentrations and HP risk was likely to be *J*-shaped generally, indicating a significant dose–effect relationship before BDG levels of 72 pg/ml and a stable high risk in the population with a slight increase after the threshold. A prospective cohort study was performed considering a confusing sequence of bacterial and fungal infections in HP to verify the relationship in the cross-sectional study. Participants with HP at the baseline were excluded in the prospective cohort study. Within 8,484.65 total person-years, HP risk was higher in the population with elevated BDG concentrations at baseline than those with low BDG concentrations. The association was confirmed with time sequence through the Cox regression model and log-rank regression. Therefore, these data suggest a relationship between elevated BDG concentrations and an increased risk of HP.

The GLB and CRP levels collected at multiple time points during the 5-year follow-up were dynamically analyzed in this prospective cohort study. The results showed that concentrations of GLB and CRP increased significantly with increasing BDG concentrations due to the inflammatory response over 5 years. The immune negative feedback regulation in response to IFI occurs because not only GLB protects humans from fungal infections, but also CRP as a routine biomarker for clinical infections rises during infection (Elluru et al., 2015; Rhedin et al., 2021). Between low and high BDG concentration, the total trend of GLB concentration was falling and the total trend of CRP concentration was rising with the increase of years. However, the changes of GLB and CRP concentration is slight during 5 years, and all above hits, which immunoregulation has undergone hidden changes after primary infection to maintain the immune homeostasis. Significantly, there were differences in the indexes such as age, TC, LDL-C, HDL-C, Apo A1, TP, GLB, and UA concentration between participants with or without HP (all *p*-value < 0.0001). The related mechanism needs to be studied on in the further.

This study has several advantages. Firstly, this is the first human-based evidence showing that the elevated BDG concentration increases the risk of HP incidence with significant dose–effect relationship no matter in men and women. Beyond of the results from cross-sectional study with a large sample size, the prospective cohort study provided longitudinal evidence which tips a potential causal relationship by time sequence. Lastly, the annual trends of GLB and CRP changes supported the conclusion above. However, there are also several limitations. Firstly, the impact of the kind of fungi on HP was not distinguished in the prospective cohort study. Secondly, the causal relationship between IFI and the elevated incidence of HP is still needed to be verified by series of method such as Mendelian analysis and so on. Besides, the relationship between IFI and the increased risk of HP was only found in Chinese in this study, and it should be further verified by ethnics in future studies. Lastly, differences in HP diagnosis rates between male and female have been found in this study, which has been not found in previous studies. And no related evidences on mechanism have been observed *in vitro* and *in vivo*, which should be verified in the future to exclude the cause of bias.

## Conclusion

In conclusion, IFI is found to increase the HP risk in population with time sequence, which hints antifungal scheme and related mechanism needed to be studied in the future as a potential prevention and treatment.

## Data Availability Statement

The original contributions presented in the study are included in the article, further inquiries can be directed to the corresponding author/s.

## Ethics Statement

This study was approved by the Ethics Committee of Xinhua Hospital Chongming Branch, affiliated with Shanghai Jiao Tong University School of Medicine. Written informed consent to participate in this study was provided by the participants’ legal guardian/next of kin.

## Author Contributions

XL and LL designed the study and revised the manuscript. LL, CL, and JRC cleaned the data and analyzed the data. LL wrote the draft of the manuscript. LL, CL, JRC, JYC, XL, and HW reviewed and edited the manuscript. JC, YYF, and KXY downloaded the original text of references. LL, CL, JRC, JYC, JC, YYF, KXY, HW, and XL have read and approved the final draft. All authors contributed to the article and approved the submitted version.

## Conflict of Interest

The authors declare that the research was conducted in the absence of any commercial or financial relationships that could be construed as a potential conflict of interest.

## Publisher’s Note

All claims expressed in this article are solely those of the authors and do not necessarily represent those of their affiliated organizations, or those of the publisher, the editors and the reviewers. Any product that may be evaluated in this article, or claim that may be made by its manufacturer, is not guaranteed or endorsed by the publisher.
